# Supplementary figures and images for: Understanding the complexity of disease-climate interactions for rice bacterial panicle blight under tropical conditions
Source: PLoS One. 2021 May 26;16(5):e0252061. doi: 10.1371/journal.pone.0252061 (PMC8153475; doi:10.1371/journal.pone.0252061)

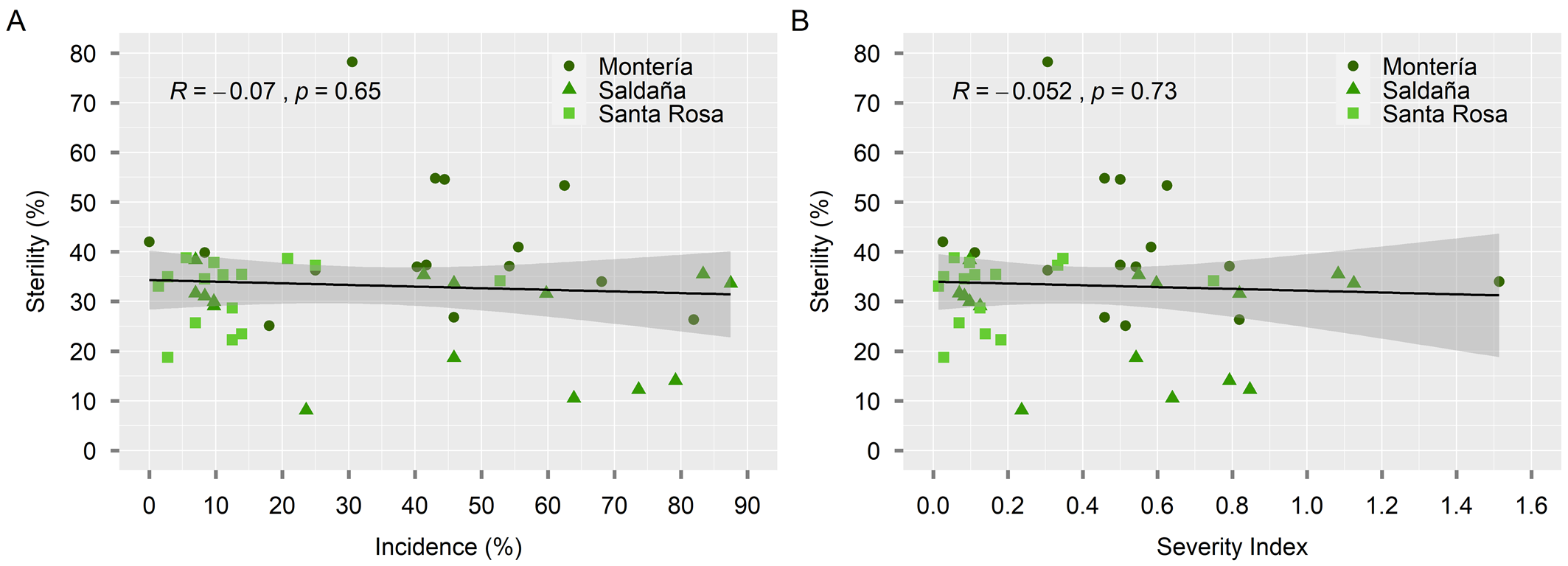

Supplement: S1 Fig — Correlation between Disease Incidence (A), Severity (B), and Spikelet Sterility in Three Locations. (TIF) [file pone.0252061.s001.tif]

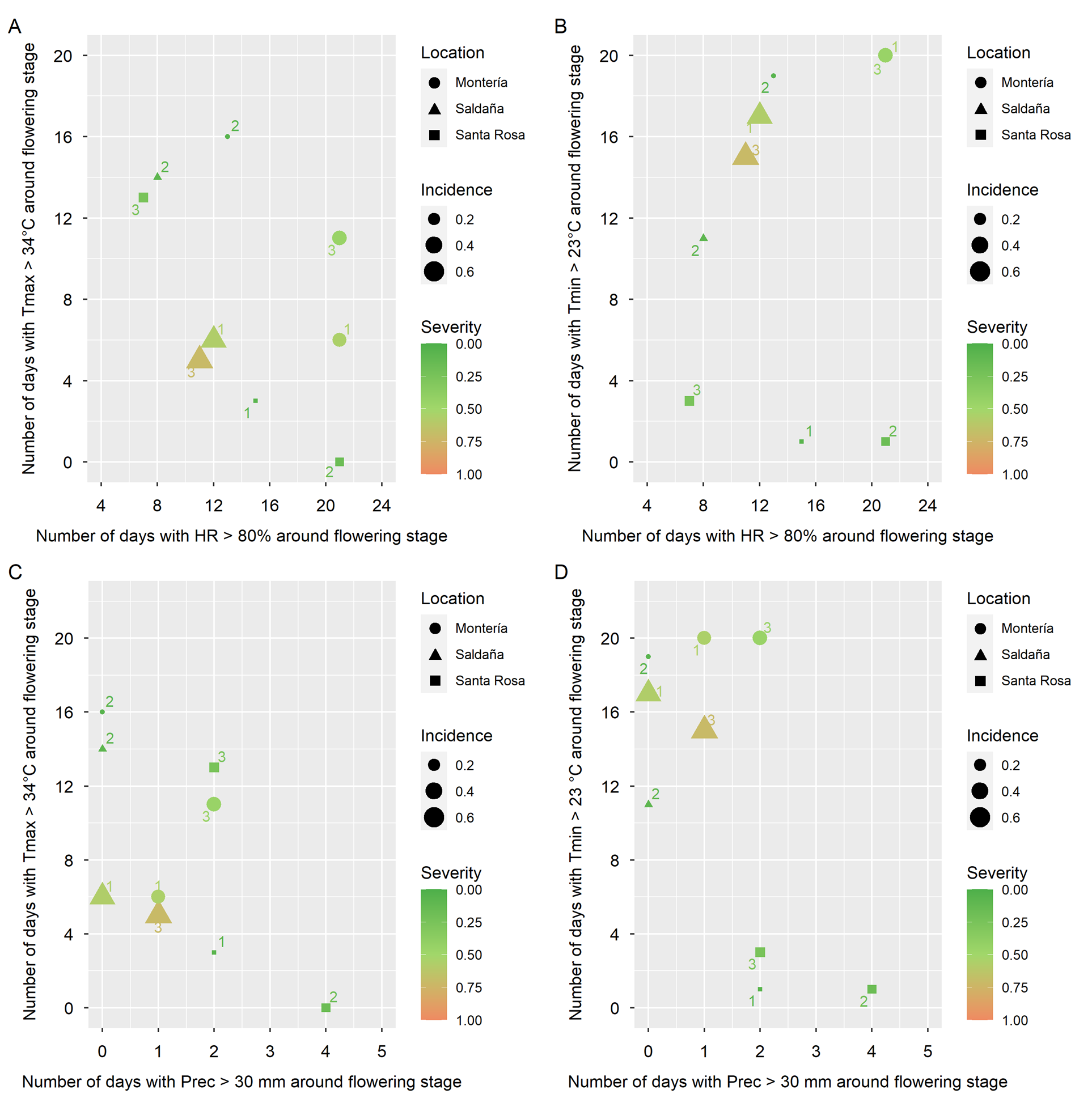

Supplement: S2 Fig — Numbers beside symbols indicate the planting date (1–3, as specified in Table 1). Panels show pairs of climate variables as follows: (A) TMAX vs precipitation; (B) TMAX vs HR; (C) TMIN vs precipitation; and (D) TMIN vs RH. All panels show severity (in colors) and sterility (symbol size). (TIF) [file pone.0252061.s002.tif]
