# Supplementary material for: Understanding the complexity of disease-climate interactions for rice bacterial panicle blight under tropical conditions
Source: PLoS One. 2021 May 26;16(5):e0252061. doi: 10.1371/journal.pone.0252061 (PMC8153475; doi:10.1371/journal.pone.0252061)
Supplement: S1 Table — (PDF) [file pone.0252061.s003.pdf]

S1 Table. Bacterial Species Used to Test P11 Primers Specificity.

Serial #	Strain Code	*Burkholderia / Paraburkholderia* species	Host/origin	DNA Source
1	STM815 ^{T}	*P. phymatum*	*Machaerium lunatum*, root nodule, french Guiana	IRD
2	ATCC25416 ^{T}	*B. cepacia*	*Allium cepa*	IRD
3	LMG21444 ^{T}	*P. tuberum*	*Aspalathus carnosa*, root nodule, South Africa	IRD
4	ATCC700544 ^{T}	*P. graminis*	Maize senescent root system, France	IRD
5	LMG19076 ^{T}	*P. caledonica*	Soil rhizosphere, Scotland	IRD
6	LMG14190 ^{T}	*B. glathei*	Lateritic soil, Germany	IRD
7	LMG20581	*P. terricola*	Agricultural soil, Belgium	IRD
8	LMG21463 ^{T}	*B. xenovorans*	PCB-contaminated soil, USA	IRD
9	PAS44 ^{T}	*P. mimosarum*	Mimosa pigra, root nodule, *Taiwan*	IRD
10	BR3407	*P. sabiae*	Mimosa caesalpiniifolia, root nodule, Brazil	IRD
11	JPY461 ^{T}	*P. diazotrophica*	*Mimosa candollei*, root nodule, Brazil	IRD
12	JPY345 ^{T}	*P. symbiotica*	*Mimosa cordistipula*, root nodule, Brazil	IRD
13	LMG16225 ^{T}	*P. fungorum*	Fungus (*Phanerochaete chrysosporium*), France	IRD
14	LMG2247 ^{T}	*P. phenazinium*	Soil enriched with threonine	IRD
15	LMG22487 ^{T}	*P. phytofirmans*	Contaminant from *Glomus vesiculiferum* - colonized onion roots	IRD
16	LMG10929 ^{T}	*B. vietnamiensis*	*Oryza sativa*, rhizosphere soil, Vietnam	IRD
17	LMG2196 ^{T}	*B. glumae*	*Oryza sativa*, grain, Japan	IRD
18	ICMP13695 ^{T}	*B. gladioli*	*Gladiolus sp.*	IRD
19	M130	*P. kururiensis*	*Oryza sativa*, Surface-sterilized roots, Brazil	IRD
20	LMG19450	*P. sacchari*	Sugar cane plantation, soil, Brazil	IRD
21	LMG22722 ^{T}	*P. unamae*	*Zea mays* rhizosphere, Mexico	IRD
22	LMG22274 ^{T}	*P. tropica*	Sugarcane, roots*, Brazil*	IRD
23	ICMP9424	*B. plantarii*	*Oryza sativa* seedling, Japan	IRD
24	3252-8	*B. glumae*	*O. sativa*	CIAT
25	4008-2	*A. avenae subsp. avenae*	*O. sativa*	CIAT

26	4500-2	*P. fuscovaginae*	*O. sativa*	CIAT
27	TG7	*Pantoea agglomerans*	O. sativa	Togo
28	PX0116	*Xanthomonas oryzae* pv. *oryzae*	*O. sativa*	CSU
29	BLS256	*Xanthomonas oryzae* pv. *oryzicola*	*O. sativa*	CSU
