# Supplementary material for: Understanding the complexity of disease-climate interactions for rice bacterial panicle blight under tropical conditions
Source: PLoS One. 2021 May 26;16(5):e0252061. doi: 10.1371/journal.pone.0252061 (PMC8153475; doi:10.1371/journal.pone.0252061)
Supplement: S2 Table — (PDF) [file pone.0252061.s004.pdf]

S2 Table. Combined Analysis of Variance for Incidence, Severity Index, and Sterility of Four Rice Genotypes across Nine Environments.

Trait	Location	Means	Source of variation						
			G	S	L	G x S	G x L	S x L	G x S x L
Incidence	Monteria	16.48 ± 1.83 b	***	***	***	***	***	***	***
	Saldaña	33.97 ± 2.50 a							
	Santa Rosa	10.94 ± 1.32 c							
Severity index	Monteria	0.34 ± 0.02 b	***	***	***	***	***	***	***
	Saldaña	0.44 ± 0.02 a							
	Santa Rosa	0.16 ± 0.02 c							
Sterility	Monteria	40.05 ± 1.41 a	***	***	***	***	***	***	***
	Saldaña	24.93 ± 1.12 c							
	Santa Rosa	30.94 ± 1.24 b							

G=Genotype; S= Season; L= location. Level of significance: *** P<0.001, ** P<0.01, * P< 0.05, ns= non- significant. Means with the same letter are not significantly different from each other (P.0.05)
