# Supplementary material for: Understanding the complexity of disease-climate interactions for rice bacterial panicle blight under tropical conditions
Source: PLoS One. 2021 May 26;16(5):e0252061. doi: 10.1371/journal.pone.0252061 (PMC8153475; doi:10.1371/journal.pone.0252061)
Supplement: S3 Table — (PDF) [file pone.0252061.s005.pdf]

S3 Table. *B. glumae* Detection on Rice Samples Collected from Field Study Using Colony Isolation and PCR.

Serial #	Sample Code	Location/Season	Colony (CFU)	PCR
83	SR2ME205	Santa Rosa 1	N	N
94	SR2F101		N	N
95	SR2F102		N	N
96	SR2F103		N	N
97	SR2F104		N	N
98	SR2F105		N	N
99	SR2F201		N	N
100	SR2F 202		N	N
101	SR2F203		N	N
102	SR2F204		N	P
103	SR2F205		N	N
104	SR2F301		N	N
105	SR2F302		N	P
106	SR2F303		N	N
107	SR2F304		N	N
108	SR2F305		N	N
109	SR2F401		N	N
110	SR2F402		N	N
111	SR2F403		N	N
112	SR2F404		N	N
113	SR2F405		N	N
114	SR2L101		5.0×10^5	P
115	SR2L102		N	N
116	SR2L103		N	N
117	SR2L104		N	P
118	SR2L105		N	N
119	SR2L201		N	N
120	SR2L202		N	N
121	SR2L203		N	N
122	SR2L204		N	N
123	SR2L205		N	N
124	SR2L301		N	N
125	SR2L302		N	N
126	SR2L303		N	N
127	SR2L304		N	N
128	SR2L305		N	N

129	SR2L401		N	N
130	SR2L402		N	N
131	SR2L403		N	N
132	SR2L404		N	P
133	SR2L405		N	N
134	SR2P102		N	N
135	SR2P103		N	N
136	SR2P104		N	N
137	SR2P105		N	P
138	SR2P201		N	N
139	SR2P202		N	N
140	SR2P203		N	N
141	SR2P204		N	N
142	SR2P301		N	N
143	SR2P302		N	N
144	SR2P304		N	N
145	SR2P305		N	N
146	SR2P401		N	N
147	SR2P402		N	N
148	SR2P403		N	N
149	SR2P405		N	P
230	M1ME101	Monteria 1	N	N
231	M1ME102		N	N
232	M1ME103		N	N
233	M1ME104		N	N
234	M1ME105		N	N
235	M1ME201		N	N
236	M1ME202		N	N
237	M1ME203		N	N
238	M1ME204		2.0×10^5	P
239	M1ME205		N	N
240	M1ME301		N	N
241	M1ME302		N	N
242	M1ME303		N	P
243	M1ME305		N	N
244	M1ME401		N	P
245	M1ME402		N	N
246	M1ME403		N	P
247	M1ME405		N	N
248	M1F101		N	N
249	M1F102		N	N
250	M1F103		N	N
251	M1F104		N	N

252	M1F105		N	N
253	M1F201		N	N
254	M1F202		N	N
255	M1F203		N	N
256	M1F204		N	P
257	M1F205		N	N
258	M1F301		N	N
259	M1F302		N	N
260	M1F303		N	N
261	M1F305		N	N
262	M1F401		N	N
263	M1F402		N	N
264	M1F403		N	N
265	M1F404		N	N
266	M1F405		N	P
267	M1L101		N	P
268	M1L102		N	N
269	M1L103		N	P
270	M1L104		N	P
271	M1L105		N	N
272	M1L201		N	N
273	M1L202		N	P
274	M1L203		N	P
275	M1L204		N	P
276	M1L205		N	N
277	M1L301		N	N
278	M1L302		N	P
279	M1L303		N	N
280	M1L305		N	P
281	M1L401		N	N
282	M1L402		N	N
283	M1L403		N	N
284	M1L404		1.0×10^{4}	N
285	M1L405		N	N
286	M1P101		N	P
287	M1P102		N	P
288	M1P103		N	P
289	M1P104		1.0×10^{5}	N
290	M1P105		N	P
291	M1P201		9.0×10^{5}	P
292	M1P202		4.0×10^{5}	P
293	M1P203		N	N

294	M1P204		2.0 x10^5	N
295	M1P205		N	N
296	M1P301		1.0 x10^5	P
297	M1P302		3.0 x10^5	P
298	M1P303		1.0 x10^5	P
299	M1P305		N	N
300	M1P401		N	N
301	M1P402		N	P
302	M1P403		N	N
303	M1P404		8.0 x10^5	N
304	M1P405		8.0 x10^5	P
305	S2ME101	Saldaña 1	N	N
306	S2ME102		N	N
307	S2ME103		N	N
308	S2ME104		N	N
309	S2ME105		N	N
310	S2ME201		N	N
311	S2ME202		N	N
312	S2ME203		N	N
313	S2ME204		N	N
314	S2ME205		N	N
315	S2ME301		N	P
316	S2ME302		N	N
317	S2ME303		N	P
318	S2ME304		N	P
319	S2ME305		N	N
320	S2ME401		N	N
321	S2ME402		N	N
322	S2ME403		N	N
323	S2ME404		N	N
324	S2ME405		N	P
325	S2F101		N	N
326	S2F102		N	N
327	S2F103		N	N
328	S2F104		N	N
329	S2F105		N	N
330	S2F201		N	N
331	S2F202		N	N
332	S2F203		N	N
333	S2F204		N	N
334	S2F205		N	N
335	S2F301		N	N

336	S2F302		N	N
337	S2F303		N	N
338	S2F304		N	N
339	S2F305		N	N
340	S2F401		N	N
341	S2F402		N	N
342	S2F403		N	N
343	S2F404		N	N
344	S2F405		N	N
345	S2L101		N	N
346	S2L102		N	N
347	S2L103		N	N
348	S2L104		N	P
349	S2L105		N	N
350	S2L201		N	N
351	S2L202		N	N
352	S2L203		N	P
353	S2L204		N	N
354	S2L205		N	N
355	S2L301		N	P
356	S2L302		N	N
357	S2L303		N	N
358	S2L304		N	N
359	S2L305		N	N
360	S2L401		N	N
361	S2L402		N	N
362	S2L403		N	N
363	S2L404		N	N
364	S2L405		N	P
365	S2P101		N	N
366	S2P102		N	N
367	S2P103		N	P
368	S2P104		N	N
369	S2P105		N	N
370	S2P201		2.4×10^{7}	N
371	S2P202		N	N
372	S2P203		N	P
373	S2P204		N	N
374	S2P205		N	N
375	S2P301		1.0×10^{5}	N
376	S2P302		N	N
377	S2P303		N	N
378	S2P304		N	N

379	S2P305		N	P
380	S2P401		N	N
381	S2P402		N	P
382	S2P403		N	N
383	S2P404		N	N
384	S2P405		N	N
385	S3ME101	Saldaña 2	N	N
386	S3ME102		N	N
387	S3ME103		N	N
388	S3ME104		N	P
389	S3ME105		N	N
390	S3ME201		N	P
391	S3ME202		N	P
392	S3ME203		N	P
393	S3ME204		N	P
394	S3ME205		N	P
395	S3ME301		N	P
396	S3ME302		N	P
397	S3ME303		N	N
398	S3ME304		N	N
399	S3ME305		N	N
400	S3ME401		N	N
401	S3ME402		N	N
402	S3ME403		2.0×10^5	N
403	S3ME404		N	N
404	S3ME405		N	N
405	S3F101		N	N
406	S3F102		N	N
407	S3F103		N	N
408	S3F104		N	P
409	S3F105		N	N
410	S3F201		N	N
411	S3F202		N	N
412	S3F203		N	N
413	S3F204		N	N
414	S3F205		N	N
415	S3F301		N	N
416	S3F302		N	N
417	S3F303		N	N
418	S3F304		N	N
419	S3F305		N	N
420	S3F401		N	N
421	S3F402		N	N

422	S3F403		N	N
423	S3F404		N	P
425	S3L101		N	P
425	S3L101		N	N
427	S3L103		N	N
428	S3L104		5.5 $x10^{6}$	P
429	S3L105		N	N
430	S3L201		N	N
431	S3L202		N	N
432	S3L203		N	N
433	S3L204		N	N
434	S3L205		N	N
435	S3L301		N	N
436	S3L302		N	N
437	S3L303		1.2 $x10^{7}$	N
438	S3L304		N	N
439	S3L305		N	N
440	S3L401		N	P
441	S3L402		N	N
442	S3L403		N	N
443	S3L404		N	N
444	S3L405		N	N
445	S3P101		N	N
446	S3P102		N	N
447	S3P103		N	N
448	S3P104		4.4 $x10^{6}$	P
449	S3P105		N	N
450	S3P201		N	P
451	S3P202		N	N
452	S3P203		N	N
453	S3P204		N	N
454	S3P205		N	P
455	S3P301		N	P
456	S3P302		N	N
457	S3P303		N	N
458	S3P304		N	P
459	S3P305		N	P
460	S3P401		N	N
461	S3P402		3.3 $x10^{7}$	N
462	S3P403		N	N
463	S3P404		N	N
464	S3P405		N	N

505	M3ME101	Monteria 2	N	N
506	M3ME102		N	P
507	M3ME103		N	P
508	M3ME104		N	N
509	M3ME105		N	N
510	M3ME201		N	P
511	M3ME202		N	P
512	M3ME203		N	P
513	M3ME204		N	P
514	M3ME205		N	P
515	M3ME301		N	N
516	M3ME302		N	N
517	M3ME303		N	P
518	M3ME304		N	N
520	M3ME401		N	P
521	M3ME402		N	P
522	M3ME403		N	P
523	M3ME404		N	P
524	M3ME405		N	P
525	M3L103		N	N
526	M3L201		N	N
527	M3L202		N	N
528	M3L203		N	N
529	M3L204		N	P
531	M3L301		N	N
532	M3L302		N	N
533	M3L303		N	N
535	M3L305		N	N
536	M3L401		N	N
537	M3L402		N	N
538	M3L403		N	P
539	M3L404		N	N
540	M3L405		N	N
541	M3P101		N	N
542	M3P102		N	N
543	M3P103		N	N
544	M3P104		N	N
545	M3P105		N	P
546	M3P201		N	N
547	M3P203		N	P
548	M3P204		N	N
550	M3P301		N	N
551	M3P302		N	N

552	M3P304		N	N
553	M3P305		8.6×10^6	N
554	M3P401		N	N
555	M3P402		N	N
556	M3P403		N	N
557	M3P404		N	N
558	M3P405		N	N
559	S4ME101	Saldaña 3	N	P
560	S4ME102		N	N
561	S4ME103		N	N
562	S4ME104		N	N
563	S4ME105		N	N
564	S4ME202		N	N
565	S4ME203		N	N
566	S4ME204		N	P
567	S4ME205		N	N
568	S4ME301		N	N
569	S4ME302		N	P
570	S4ME303		N	N
571	S4ME304		N	N
572	S4ME305		N	N
573	S4ME401		N	N
574	S4ME402		N	N
575	S4ME403		N	P
576	S4ME404		N	N
577	S4ME405		N	N
578	S4F101		N	N
579	S4F102		N	N
580	S4F103		N	N
581	S4F104		N	N
582	S4F105		N	N
583	S4F202		N	N
584	S4F203		N	N
585	S4F204		N	N
586	S4F205		N	N
587	S4F301		N	N
588	S4F302		N	P
589	S4F303		N	N
590	S4F304		N	P
591	S4F305		N	N
592	S4F401		N	P
593	S4F402		N	N
594	S4F403		N	N

595	S4F404		N	N
596	S4F405		N	N
597	S4L101		N	N
598	S4L102		N	N
599	S4L103		N	N
600	S4L104		N	N
601	S4L105		N	P
602	S4L202		N	P
603	S4L203		N	P
604	S4L204		N	N
605	S4L205		N	P
606	S4L301		N	N
607	S4L302		N	P
608	S4L303		N	N
609	S4L304		N	N
610	S4L305		N	P
611	S4L401		N	P
612	S4L402		N	N
613	S4L403		N	P
614	S4L404		N	P
615	S4L405		N	N
617	S4P101		N	N
618	S4P102		N	N
619	S4P103		N	N
620	S4P104		1.8×10^6	N
621	S4P105		N	P
622	S4P202		N	N
623	S4P203		N	N
624	S4P204		N	N
625	S4P205		N	P
626	S4P301		N	N
627	S4P302		N	N
628	S4P303		N	P
629	S4P304		N	N
630	S4P305		N	P
631	S4P401		6.0×10^5	P
632	S4P402		N	P
633	S4P403		N	N
634	S4P404		N	P
635	S4P405		N	N
636	SR3ME101	Santa Rosa 2	N	N
637	SR3ME102		N	N
638	SR3ME103		N	N

639	SR3ME104		N	N
640	SR3ME105		N	N
641	SR3ME201		N	N
643	SR3ME203		N	N
645	SR3ME205		N	N
646	SR3ME301		N	N
647	SR3ME302		N	N
648	SR3ME303		N	N
649	SR3ME304		N	N
650	SR3ME305		N	N
651	SR3ME401		N	N
652	SR3ME402		N	N
653	SR3ME403		N	N
654	SR3ME404		N	N
655	SR3ME405		N	N
656	SR3F101		N	N
657	SR3F102		N	N
658	SR3F103		N	N
659	SR3F104		N	N
660	SR3F105		N	N
661	SR3F201		N	N
662	SR3F202		N	N
663	SR3F203		N	N
664	SR3F204		N	N
665	SR3F205		N	N
666	SR3F301		N	N
667	SR3F302		N	N
668	SR3F303		N	N
669	SR3F304		N	N
670	SR3F305		N	N
671	SR3F401		N	N
672	SR3F402		N	N
673	SR3F403		N	N
674	SR3F404		N	N
675	SR3F405		N	N
676	SR3L101		N	N
677	SR3L102		N	N
678	SR3L103		N	N
679	SR3L104		N	N
680	SR3L105		N	N
681	SR3L201		N	N
682	SR3L202		N	N
683	SR3L203		N	N

684	SR3L204		N	N
685	SR3L205		N	P
686	SR3L301		N	N
687	SR3L302		N	N
688	SR3L303		N	N
689	SR3L304		N	N
690	SR3L305		N	N
691	SR3L401		N	N
692	SR3L402		N	N
693	SR3L403		N	N
694	SR3L404		N	N
695	SR3L405		N	N
696	SR3P101		N	N
697	SR3P102		N	N
698	SR3P103		N	N
699	SR3P104		N	P
700	SR3P105		N	P
701	SR3P201		N	N
702	SR3P202		N	N
703	SR3P203		N	N
704	SR3P204		N	N
705	SR3P205		N	P
706	SR3P301		N	N
707	SR3P302		N	N
708	SR3P303		N	P
709	SR3P304		N	P
710	SR3P305		N	N
711	SR3P401		N	N
712	SR3P402		N	N
713	SR3P403		N	P
714	SR3P404		N	P
715	SR3P405		N	P
716	M4ME101	Monteria 3	N	N
717	M4ME102		N	N
718	M4ME103		N	N
719	M4ME104		N	N
720	M4ME105		N	N
721	M4ME201		N	N
722	M4ME202		N	P
723	M4ME203		N	N
724	M4ME204		N	N
725	M4ME205		N	N
726	M4ME301		N	N

727	M4ME302		N	N
728	M4ME303		N	N
729	M4ME304		N	N
730	M4ME305		N	N
731	M4ME401		N	N
732	M4ME402		N	N
733	M4ME403		N	N
734	M4ME404		N	N
735	M4ME405		N	N
736	M4F101		N	N
737	M4F102		N	N
738	M4F103		N	N
739	M4F104		N	N
740	M4F105		N	N
741	M4F201		N	P
742	M4F202		N	P
743	M4F203		N	P
744	M4F204		N	N
745	M4F205		N	N
746	M4F301		N	N
747	M4F302		N	N
748	M4F303		N	N
749	M4F304		N	N
750	M4F305		N	N
751	M4F401		N	P
752	M4F402		N	N
753	M4F403		N	N
754	M4F404		N	N
755	M4F405		N	N
756	M4L101		N	P
757	M4L102		N	N
758	M4L103		N	P
759	M4L104		N	N
760	M4L105		N	N
761	M4L201		N	P
762	M4L202		N	N
763	M4L203		N	N
764	M4L204		N	P
765	M4L205		N	P
766	M4L301		N	N
767	M4L302		N	N
768	M4L303		N	P
769	M4L304		N	N

770	M4L305		N	N
771	M4L401		N	N
772	M4L402		N	N
773	M4L403		N	N
774	M4L404		N	P
775	M4L405		N	P
776	M4P101		N	N
777	M4P102		N	P
778	M4P103		N	P
779	M4P104		N	N
780	M4P105		N	P
781	M4P201		N	N
782	M4P202		N	P
783	M4P203		N	N
784	M4P204		N	P
785	M4P205		N	P
786	M4P301		N	P
787	M4P302		N	N
788	M4P303		N	P
789	M4P304		N	P
790	M4P305		N	N
791	M4P401		N	N
792	M4P402		N	P
793	M4P403		N	N
794	M4P404		N	P
795	M4P405		N	P
796	SR4ME101	Santa Rosa 3	N	N
797	SR4ME102		N	N
798	SR4ME103		N	N
799	SR4ME104		N	N
800	SR4ME105		N	N
801	SR4ME201		N	P
802	SR4ME202		N	N
803	SR4ME203		N	N
804	SR4ME204		N	N
805	SR4ME205		N	N
806	SR4ME301		N	N
807	SR4ME302		N	P
808	SR4ME303		N	N
809	SR4ME304		N	P
810	SR4ME305		N	N
811	SR4ME401		N	N
812	SR4ME402		N	N

813	SR4ME403		4.0 x10^5	N
814	SR4ME404		N	N
815	SR4ME405		N	N
816	SR4F101		N	N
817	SR4F102		N	N
818	SR4F103		N	N
819	SR4F104		N	N
820	SR4F105		N	N
821	SR4F201		N	N
822	SR4F202		N	N
823	SR4F203		N	N
824	SR4F204		N	N
825	SR4F205		N	N
826	SR4F301		N	N
827	SR4F302		N	N
828	SR4F303		N	N
829	SR4F304		N	N
830	SR4F305		N	N
831	SR4F401		N	N
832	SR4F402		N	N
833	SR4F403		N	N
834	SR4F404		N	N
835	SR4F405		N	N
836	SR4L101		N	N
837	SR4L102		N	N
838	SR4L103		N	N
839	SR4L104		N	N
840	SR4L105		N	N
841	SR4L201		N	N
842	SR4L202		N	N
843	SR4L203		N	N
844	SR4L204		N	N
845	SR4L205		N	N
846	SR4L301		N	N
847	SR4L302		N	N
848	SR4L303		N	N
849	SR4L304		N	N
850	SR4L305		N	N
851	SR4L401		N	N
852	SR4L402		N	N
853	SR4L403		N	N
854	SR4L404		N	N
855	SR4L405		N	N

856	SR4P101		N	N
857	SR4P102		N	N
858	SR4P103		N	N
859	SR4P104		N	N
860	SR4P105		N	N
861	SR4P201		N	N
862	SR4P202		N	N
863	SR4P203		N	N
864	SR4P204		N	N
865	SR4P205		N	N
866	SR4P301		N	N
867	SR4P302		N	N
868	SR4P303		N	N
869	SR4P304		N	N
870	SR4P305		N	N
871	SR4P401		N	N
872	SR4P402		N	N
873	SR4P403		N	N
874	SR4P404		N	N
875	SR4P405		N	N

ME= Booting stage samples

F= Flowering stage samples

L= Milky stage samples

P= Dough stage samples

N= Negative result

P= Positive result
