# Supplementary material for: Understanding the complexity of disease-climate interactions for rice bacterial panicle blight under tropical conditions
Source: PLoS One. 2021 May 26;16(5):e0252061. doi: 10.1371/journal.pone.0252061 (PMC8153475; doi:10.1371/journal.pone.0252061)
Supplement: S4 Table — (PDF) [file pone.0252061.s006.pdf]

S4 Table. Climate Conditions Registered during 21 Days' Time Frame during Flowering Time.

Location	Season	# of Days RH>80%	# of Days TMAX >34° C	# of Days TMIN >23° C	# of Days RAIN >30mm
**Santa Rosa**	1	15	3	1	2
	2	21	0	1	4
	3	7	13	3	2
**Saldaña**	1	12	6	17	0
	2	8	14	11	0
	3	11	5	15	1
**Montería**	1	21	6	20	1
	2	13	16	19	0
	3	21	11	20	2

RH, relative humidity

TMAX, maximum temperature

TMIN, minimum temperature
