# Supplementary material for: Understanding the complexity of disease-climate interactions for rice bacterial panicle blight under tropical conditions
Source: PLoS One. 2021 May 26;16(5):e0252061. doi: 10.1371/journal.pone.0252061 (PMC8153475; doi:10.1371/journal.pone.0252061)
Supplement: Attachment — Submitted filename: Responsetoreviewers.docx [file pone.0252061.s007.docx]

**Response to reviewers PONE-D-21-01039
Understanding the Complexity of Disease-Climate interactions for Rice Bacterial Panicle Blight under Tropical Conditions**

Dear editor and reviewers, thank you so much for taking the time to revise our manuscript and also the good comments about our research. We revised the manuscript very carefully, added new information, and eliminated some content according to the main comments you did.

In the flowing sections, you will find answers to the main comments.

Best regards,

Gloria Mosquera and co-authors.

**Reviewer 1**

Reviewer comment:

My main concern with this study is related to the analysis of the presence of the pathogen using PCR. The study tries to provide explanation on disease parameters based on their ability to detect bacteria by PCR. PCR is not appropriate to do that due to technical limitations extracting DNA from particular tissues or genotypes, or the fact that PCR can generate a false positive, amplifying DNA from dead bacteria that are not actively contribute to infection. The best test to determine active infection is by isolation and quantification of bacteria. In addition, the text does not clearly explain (I apologize if I missed it), which gene was used to design PCR primers. Is it really B. glumae specific? There are many species of Burkholderia in natural environments.

R/. B. glumae presence on plant tissue should be consider as part of colonization since this specie is not a endophytic nor saprofitic bacterial on rice. It is a true pathogen. Under this mode of interaction with the host we can assume that even if the bacteria is dead at the moment of the test, it was there because of an infection. We totally agree with the comment if the test is done at harvesting time or postharvest. Since bacterial detection on completely matured seeds cannot be claimed as potential cause of any damage during initial panicle development and grain filling process.

*B. glumae* morphology could be diverse and it has been documented in the literature. This feature makes bacterial counting inaccurate since it relies on the expertise of the Lab personnel, for this reason, we used selective media and confirm suggestive bacteria by colony PCR. We simultaneously analyzed all 655 samples for bacterial isolation and plant- DNA PCR test. Since a big proportion of the samples were negative for both methods, we decided to include only plant tissue PCR data in the original manuscript because we consider that this technique can be more accurate and fast that media growth.

The new primers used in our study were designed by our group. The most relevant information about the primers was added to the manuscript. We are confident about PCR methodology and primers described here since as you may see in the results section, PCR assays were carried out following high standard procedures for specificity and sensitivity, with the inclusion of positive and negative controls. In addition, we also carried out bacterial isolation and quantitation from the same samples. As is documented in the results, this method yielded less positive samples, compared to direct PCR. We hope this information would be helpful to respond reviewer comments.

Reviewer comment:

In addition, the study makes grandiose interpretations when the percentage of positive results is very low, and the experiments lack appropriate controls to determine if the results are biologically meaningful, or associated with technical problems. For example, statements such as: “Disaggregated analysis by location evidenced Montería as the site with the highest number of infected samples and where the bacterial infection along three different seasons was quite stable” (Line361-363), begs the question, what is the criteria to consider that scenario “stable”, just the ability to detect bacteria? The levels of detection of bacteria are too low to support such a bold statement.

**R/ We were not sure which kind of “appropriate controls” the reviewer is referring to. If is referred to controls for the PCR test, sensitivity test provided evidence about the reliability of the method to detect *B. glumae* on seeds. Another kind of control for the field experiments is the cultivar Fedearroz 2000, we include information about the known susceptibility of this genotype to the infection by *B. glumae*.**

**We remove the content related to disaggregated data on bacterial detection on each panicle stage because indeed, the infection level was low in all the samples. However, Monteria and Saldaña indeed were places with more infected samples compared to Santa Rosa. We used the term stable to described the fact that all the three planting dates yielded infected samples, we have observed that sometimes the disease disappears in the field from one cycle to another. This is one of the main problems for breeders to select resistant germplasm.**

Line 371: States “PCR results were used to elucidate the dynamic of bacterial infection through panicle development”. That statement indicates a lot more than what the data actually shows. The data exclusively shows sampling at different developmental stages, does not necessarily provides mechanistic insight into the “dynamics of bacterial infection”. Dynamics of infection will mean having the ability to monitor disease progression by tracking bacteria throughout the tissues, which is not what they did.
Since the field data is solid and well analyzed and the PCR data does not add any particular insight, I would recommend remove all the PCR data.

**R/. We agree with the comment and this information was removed from the manuscript.**

**Minor comments were fully addressed.**

Reviewer #2

Reviewer comment:

1. The Results part needs an elaborated editing process to make sentences shorter and clearer. Overall, it tends to be too lengthy describing too much details of data presented in Table 2. English writing itself also looks rough in the Results part compared with other parts including introduction, so additional round of internal review process is recommended.

**R/ Section describing previous Table 3 was trimmed a little bit. English writing was revised and edited by a native English scientific editor. We hope the language on this new version fulfill the quality required by the journal.**

**Minor comments were also addressed.**
