# Supplementary material for: Understanding the complexity of disease-climate interactions for rice bacterial panicle blight under tropical conditions
Source: PLoS One. 2021 May 26;16(5):e0252061. doi: 10.1371/journal.pone.0252061 (PMC8153475; doi:10.1371/journal.pone.0252061)
Supplement: Attachment — Submitted filename: Response to Reviwers.docx [file pone.0252061.s008.docx]

May 4, 2021

Dear Editor and Reviewers

PLOS ONE Journal

REF: PONE-D-21-01039R1

We are very thankful to receive positive feedback in your second round of revision on the manuscript “Understanding the Complexity of Disease-Climate Interactions for Rice Bacterial Panicle Blight under Tropical Conditions” by Echeverri *et al*. At the same time, we went through all your suggestions to fulfill the requirements for acceptance. You will see marked editions along the document according to all the minor comments suggested by both reviewers, in addition, we also edited other errors we detected during the revision process. Special attention was put on the discussion section, where more information and deeper description of the results were needed to cover your valid concerns. As part of it, we rectified information about yield loss published by Echeverri et al, 2019, in the previous version we referenced unintended mistaken percentage values for Fedearroz 2000 and Fedearroz 473. We also added a reference (Delerce et al) about climate and yield study done in Saldaña because we consider it was necessary to cite these results to clarify the point about other factors impacting yield in the region. In addition to our results, that study help us to construct a solid base for concluding that the use of severity is the most recommended parameter to establish correlation between *B. glumae* and yield loss. Other wording changes were also introduced in the discussion, Lines 454 to 507, track changes document.

The following comment from reviewer #3 *“L366-373: Although the overall number of positive samples was low, was there any indication that a particular sampling timepoint produced more positive samples than others? Perhaps pathogen presence was too low at the earlier sampling dates, but was sufficient at later dates/stages?”* was not addressed in the document. But here is the response to your question. In the first submitted version, we described in detail how many samples of each developmental stage tested positive for B. glumae. Results showed that in certain sites/seasons infected samples were higher at the pre-flowering stage, but in others, higher numbers corresponded to milky and dough stages. Results did not show a specific trend, which was the reason why one of the reviewer suggested that the information did not provide enough evidence for suggesting infection dynamics related to panicle development. In the later version we removed this information.

We hope this version will cover all your expectations about our research, results and conclusions.

Best regards,

Gloria M. Mosquera (corresponding author).
